# Elevated Genetic Diversity in the Emerging Blueberry Pathogen *Exobasidium maculosum*

**DOI:** 10.1371/journal.pone.0132545

**Published:** 2015-07-24

**Authors:** Jane E. Stewart, Kyle Brooks, Phillip M. Brannen, William O. Cline, Marin T. Brewer

**Affiliations:** 1 Department of Plant Pathology, University of Georgia, Athens, Georgia, United States of America; 2 Department of Plant Pathology, North Carolina State University, Horticultural Crops Research Station, Castle Hayne, North Carolina, United States of America; University of California-Riverside, UNITED STATES

## Abstract

Emerging diseases caused by fungi are increasing at an alarming rate. Exobasidium leaf and fruit spot of blueberry, caused by the fungus *Exobasidium maculosum*, is an emerging disease that has rapidly increased in prevalence throughout the southeastern USA, severely reducing fruit quality in some plantings. The objectives of this study were to determine the genetic diversity of *E*. *maculosum* in the southeastern USA to elucidate the basis of disease emergence and to investigate if populations of *E*. *maculosum* are structured by geography, host species, or tissue type. We sequenced three conserved loci from 82 isolates collected from leaves and fruit of rabbiteye blueberry (*Vaccinium virgatum*), highbush blueberry (*V*. *corymbosum*), and southern highbush blueberry (*V*. *corymbosum* hybrids) from commercial fields in Georgia and North Carolina, USA, and 6 isolates from lowbush blueberry (*V*. *angustifolium*) from Maine, USA, and Nova Scotia, Canada. Populations of *E*. *maculosum* from the southeastern USA and from lowbush blueberry in Maine and Nova Scotia are distinct, but do not represent unique species. No difference in genetic structure was detected between different host tissues or among different host species within the southeastern USA; however, differentiation was detected between populations in Georgia and North Carolina. Overall, *E*. *maculosum* showed extreme genetic diversity within the conserved loci with 286 segregating sites among the 1,775 sequenced nucleotides and each isolate representing a unique multilocus haplotype. However, 94% of the nucleotide substitutions were silent, so despite the high number of mutations, selective constraints have limited changes to the amino acid sequences of the housekeeping genes. Overall, these results suggest that the emergence of Exobasidium leaf and fruit spot is not due to a recent introduction or host shift, or the recent evolution of aggressive genotypes of *E*. *maculosum*, but more likely as a result of an increasing host population or an environmental change.

## Introduction

Many emerging plant diseases are caused by pathogens that have found new hosts or have been introduced to new areas, often as a result of human-mediated movement [1]. Other plant diseases are emerging as a result of the evolution of more aggressive genotypes of the pathogen [2], or genotypes that have evolved to overcome host resistance [3] or resist fungicides or antibiotics [4,5]. However, emerging diseases can also be the result of environmental changes that impact the pathogen’s habitat, including changes in climate or host populations [6]. Population genetic studies can elucidate whether emerging pathogens are introduced or native. Invasive populations will often show signatures of a population bottleneck with a reduction in genetic diversity compared to the source population due to founder effects [7,8]. However, a loss of genetic diversity can be moderated by multiple introductions [9,10]. Additionally, pathogens that emerge as the result of a recent adaptation, such as fungicide resistance, often exhibit a reduction in genetic diversity due to positive selection acting on one or a few genotypes that spread rapidly [11]. Conversely, emerging pathogens that result from changing environmental conditions or expanding host populations may not show a reduction in genetic diversity [12], and show signatures of recent population expansions. Causes for disease emergence can be understood by establishing the demographic history and genetic diversity of emerging pathogen populations [13].

Exobasidium leaf and fruit spot of blueberry is an emerging disease that has rapidly increased in prevalence since 2011 throughout the southeastern USA in the commercial blueberry production regions of Georgia, North Carolina, and Mississippi [14,15]. The disease occurs on rabbiteye blueberry (*Vaccinium virgatum*), highbush blueberry (*V*. *corymbosum*) and southern highbush blueberry (*V*. *corymbosum* hybrids), but cultivars of rabbiteye blueberry seem to be the most susceptible. The fruit stage of the disease can result in losses of 60–70% in some plantings [16]. The causal pathogen was recently described as a new species, *Exobasidium maculosum* [14]. Although Exobasidium leaf and fruit spot was first documented in the southeastern USA on highbush blueberry in North Carolina in 1997 [16], it was sporadic and occurred in isolated fields. The factors leading to emergence of this disease in the southeastern USA are not clear. However, a similar Exobasidium leaf spot disease was reported in 1997 on lowbush blueberry (*V*. *angustifolium*) in Nova Scotia, Canada [17], and Burt [18] described *Exobasidium* on lowbush blueberry in New Brunswick, suggesting the presence of the pathogen in northeast North America for at least 100 years [17]. However, *E*. *maculosum* populations from the southeastern USA are genetically distinct from populations from lowbush blueberry in northeastern North America based on the large subunit of the rDNA (LSU) [14]; but, reciprocal monophyly between populations, which would provide support for distinct species based on a phylogenetic species recognition concept [19], was not observed. Sequences from additional loci are necessary to use genealogical concordance to identify if these are distinct species or genetically different populations of the same species. Cross-inoculation studies with the genetically distinct populations onto different *Vaccinium* hosts have not been successful due to difficulty in reproducing disease symptoms [14], so it is difficult to determine if these populations are host specialized or geographically isolated.

The genus *Exobasidium* includes a diverse group of biotrophic plant pathogens that infect members of the Ericales, including *Symplocus*, *Rhododendron*, *Camellia*, and *Vaccinium*, including wild and commercial blueberry and cranberry species within *Vaccinium* section Cyanococcus. *Exobasidium* species cause a variety of plant deformities including localized leaf and fruit spots, systemic shoot infections characterized by reddened leaves, or tumors (galls) on leaves, stems, flowers, shoots, and buds [16,18,20–22]. Infections can be annual or perennial and local or systemic, with both types of infections occurring on blueberry and cranberry. A felt-like layer that forms on the underside of leaves or on the entire surface of plant organs is an exposed hymenium where the sexual reproductive structures (basidia) and sexually produced spores (basidiospores) are produced. Most members of the genus are dimorphic with a yeast-like growth form in culture and hyphal growth inside plant tissues [23]. Asexual reproduction occurs when yeast-like conidia bud from basidiospores or other conidia. However, basidiospores of some *Exobasidium* species germinate to produce hyphae in culture [21]. *E*. *maculosum* produces annual, localized infections that are characterized by chlorotic, circular, and sunken lesions with white, felt-like spots on the abaxial side. Basidiospores are produced from the felt-like hymenial layer. In culture, basidiospores of *E*. *maculosum* bud to form abundant yeast-like conidia [14]. Currently, the lifecycle of *E*. *maculosum* and the disease cycle of Exobasidium leaf and fruit spot of blueberry are not well understood.

Our goals are to understand why Exobasidium leaf and fruit spot is emerging in the southeastern USA, to identify factors that may be driving population divergence, and to determine if *E*. *maculosum* in the southeastern USA represents a different population or a distinct species from the isolates causing disease on lowbush blueberry in northeastern North America. The objectives of this study were to use a multilocus sequencing approach to estimate genetic diversity in *E*. *maculosum* to characterize demographic processes (founder effect vs. population expansion) underlying disease emergence, and to determine if populations are structured based on geography, blueberry host species, or host tissue (leaf or fruit) type.

## Materials and Methods

### Fungal isolation and cultures

Symptomatic leaf and fruit tissues infected with *E*. *maculosum* were collected from cultivars of rabbiteye blueberry (*V*. *virgatum*), highbush blueberry (*V*. *corymbosum*) and southern highbush blueberry (*V*. *corymbosum* hybrids) from commercial fields in Georgia and North Carolina, USA. Isolates of *Exobasidium* causing leaf spot on lowbush blueberry (*V*. *angustifolium*) and isolates of *E*. *rostrupii* causing leaf spot on cranberry (*V*. *macrocarpon*) were obtained from infected leaves sent from Maine and New Jersey, USA, respectively. Leaf spots were excised with a sterile scalpel and tissue was affixed to the lid of a Petri dish with petroleum jelly, allowing basidiospores to fall or eject onto potato dextrose agar (PDA). After 8–12 hrs incubation in the dark at 23°C, single basidiospores were collected using a sterile dissecting needle and transferred to a new dish of PDA. Fruit spots were excised and surface sterilized in 10% sodium hypochlorite, plated on PDA, and incubated in the dark at 23°C. Single colony forming units likely derived from a single conidium (yeast cell) of *Exobasidium* isolates were obtained using a quadrant streaking protocol [24]. Additional isolates of *Exobasidium* causing leaf spot on lowbush blueberry in Nova Scotia, Canada were obtained from the collection of Nancy Nickerson (Atlantic Food and Horticulture Research Centre, Kentville, Nova Scotia, Canada). For this study there were 82 isolates of *E*. *maculosum* from blueberry from the southeastern USA (Georgia and North Carolina), 6 isolates of *Exobasidium* from lowbush blueberry from northeastern North America (Maine and Nova Scotia), and an isolate of *E*. *rostrupii* from cranberry for use as an outgroup for phylogenetic analyses (Table 1). Field studies were conducted on private land with permission granted from the owner or public land where permission was not required because our studies did not involve endangered or protected species. All isolates were stored in 30% glycerol at -80°C.

**Table 1:** Original *Vaccinium* host, tissue type, and geographic location for *Exobasidium maculosum* and *E*. *rostrupii* isolates.

*Exobasidium* species^a^	*Vaccinium* host of origin^b^	Tissue	Location	Isolates (total number)
*E*. *maculosum*	*V*. *virgatum* (R) Premier	leaf	Bacon Co., GA	A1-1, A1-2, A1-3, A1-4, A2-13, A3-2, A3-3, A3-4, A4-1, A4-2, A5-1, A5-2, A5-3, A5-15, A6-17, A6-2, A6-4, A8-1 D2-6, D2-8, D5-2, D2-7 (22)
*E*. *maculosum*	*V*. *virgatum* (R) Climax	leaf	Bacon Co., GA	A7-3, A7-4, E3-1 (3)
*E*. *maculosum*	*V*. *virgatum* (R) Tifblue	leaf	Bacon Co., GA	B2, E1-1, E1-2, E1-3, E1-7 (5)
*E*. *maculosum*	*V*. *virgatum* (R) Brightwell	leaf	Bacon Co., GA	B24, B26, B28, B3, B31, E4-4, E4-1 (7)
*E*. *maculosum*	*V*. *virgatum* (R) Woodard	leaf	Bacon Co., GA	E2-2, E2-5 (2)
*E*. *maculosum*	*V*. *virgatum* (R) Powder Blue	leaf	Bacon Co., GA	E5-2, E5-3, D1-1, D1-2, D1-5 (5)
*E*. *maculosum*	*V*. *virgatum* (R) Premier	leaf	Coffee Co., GA	C1-1, C1-14, C1-16, C1-2, C1-4, C2-1, C2-16, C2-4, C3-2, C3-3, C3-4, C4-1, C4-16 (13)
*E*. *maculosum*	*V*. *virgatum* (R) Premier	fruit	Clinch Co., GA	FP1-1 (1)
*E*. *maculosum*	*V*. *virgatum* (R) Premier	fruit	Ware Co., GA	FP2-2 (1)
*E*. *maculosum*	*V*. *corymbosum* hybrid (S) Star	leaf	Bacon Co., GA	E6-2 (1)
*E*. *maculosum*	*V*. *corymbosum* hybrid (S) Star	fruit	Bacon Co., GA	FS2-1, FS2-4, FS2-8, FS2-9, FS3-1, FS3-2, FS3-2, FS2-9 (8)
*E*. *maculosum*	*V*. *virgatum* (R) Columbus	leaf	Sampson Co., NC	NCLC1-22, NCLC1-23 (2)
*E*. *maculosum*	*V*. *virgatum* (R) Powder Blue	leaf	Sampson Co., NC	NCLC1-37 (1)
*E*. *maculosum*	*V*. *virgatum* (R) Premier	leaf	Sampson Co., NC	NCPC1-1, NCPC1-2, NCPC1-8 (3)
*E*. *maculosum*	*V*. *corymbosum* hybrid (S) Legacy	leaf	Sampson Co., NC	NCLC1-14, NCLC1-15 (2)
*E*. *maculosum*	*V*. *corymbosum* hybrid (S) Blue Ridge	leaf	Sampson Co., NC	NCLC1-33, NCLC1-35 (2)
*E*. *maculosum*	*V*. *corymbosum* hybrid (S) Star	fruit	Sampson Co., NC	FS1-10 (1)
*E*. *maculosum*	*V*. *corymbosum* (H) Duke	leaf	Sampson Co., NC	NCLC1-41, NCLC1-44, NCLC1-45 (3)
*E*. maculosum*	*V*. *angustifolium* (L)	leaf	Kings Co., Nova Scotia	E86-1, E88-9, E89-5, E92-26 (4)
*E*. maculosum*	*V*. *angustifolium* (L)	leaf	Lunenburg Co., Nova Scotia	E92-50 (1)
*E*. maculosum*	*V*. *angustifolium* (L)	leaf	Waldo Co., ME	ME1-13 (1)
*E*. *rostrupii*	*V*. *macrocarpon* (C)	leaf	Burlington Co., NJ	CNJ1-1 (1)

^a^ Isolates from lowbush blueberry marked by an asterisk (*) were previously identifed as *Exobasidium* sp. A (14). Isolates from Nova Scotia were collected by N. Nickerson and kindly provided by P. Hildebrand, Atlantic Food and Horticulture Research Centre, Kentville, Nova Scotia, Canada. The isolate from Maine was obtained from an infected leaf kindly provided by S. Annis, University of Maine, Orono, ME. Isolates of *E*. *rostrupii* from cranberry were obtained from infected leaves kindly provided by J. Polashock, USDA-ARS, Genetic Improvement of Fruits and Vegetables Laboratory, Chatsworth, NJ.

^b^ Species include: *V*. *virgatum* or rabbiteye blueberry (R), *V*. *corymbosum* hybrid or southern highbush blueberry (S), *V*. *corymbosum* or highbush blueberry (H), *V*. *angustifolium* or lowbush blueberry (L), and *V*. *macrocarpon* or cranberry (C). Cultivar is listed, if known.

### Genomic DNA isolation, sequencing and alignment

Genomic DNA was extracted from yeast colonies grown on PDA for 10 to 14 days at 23°C in the dark following a standard protocol for fresh fungal tissue [25] or using a DNeasy Plant Mini Kit following the manufacturer’s protocol (Qiagen Sciences). Using the standard protocol, approximately 80 to 100 mg (fresh weight) of yeast cells were incubated in lysis buffer (50 mm EDTA pH 8, 100 mm Tris pH 8, 3.5% SDS, 250 μg/mL proteinase K, 1% sodium bisulfate) for 15 min at 65°C. After centrifugation for 5 min at 12,200 rpm, 200 μL 7.5 m ammonium acetate was added to the supernatant, vortexed, placed on ice for 15 min, then centrifuged again for 3 min. The supernatant was precipitated with isopropanol and the pellet was rinsed twice with 70% ethanol, dried and resuspended in H_2_O.

Three nuclear loci were PCR amplified and sequenced from each isolate. Regions sequenced included the internal transcribed spacer region (ITS) of the ribosomal DNA, elongation factor 1-alpha (*EF-1α*), and calmodulin (*CAL*). For ITS, the primers used were ITS1 (5'- TCCGTAGGTGAACCTGCGG-3') and ITS4 (5'-TCCTCCGCTTATTGATATGC-3') [26]; for *EF-1α*, the primers used were EF1-728F (5'-CATCGAGAAGTTCGAGAAGG-3') and EF1-986R (5'-TACTTGAAGGAACCCTTACC-3') [27]; and for *CAL*, the primers used were CAL-228F (5'- GAGTTCAAG GAGGCCTTCTCC C -3') and CAL-737R (5'-CATCTTTCTGGCCATCATGG-3') [27]. The PCR reactions were carried out in a total volume of 25 μL. Reaction components included 2.5 μL of 10× PCR buffer (Takara Bio, Inc.), 2.5 μL dNTPs, 1.25 μL of 10 μM forward and reverse primers, 0.15 μL ExTaq (Takara Bio, Inc.), and 1 μL DNA template. Cycling conditions included an initial denaturation step at 94°C for 2 minutes followed by 40 cycles with a denaturation step at 94°C for 30 s, annealing at 55°C (*EF-1α* at 60°C) for 30 s, extension at 72°C for 5 min, followed by a final extension at 72°C for 5 min. PCR products were purified with QIAquick spin columns (Qiagen Sciences). All DNA fragments were sequenced at the Georgia Genomics Facility, University of Georgia, Athens, GA using the Applied Biosystems Automated 3730 DNA Analyzer with Big Dye Terminator chemistry and Ampli-Taq-FS DNA Polymerase. All gene regions were sequenced in both directions. Sequences were aligned and manually edited in Geneious v6 (Biomatters, Inc.). There was a large amount of sequence variation among isolates so we tested the consistency of our sequencing results by randomly choosing six isolates for resequencing and comparison with the initial results. Sequences of ITS, *EF-1α*, and *CAL* were deposited in GenBank under accession numbers KR262338-KR262425, KR262258-KR262337, and KR262175-KR262257, respectively.

### Identification of conserved regions, coding regions, and synonymous and nonsynonymous substitutions

For *E*. *maculosum* and *E*. *rostrupii* sequences, the conserved 5.8S region of the ITS rDNA and coding regions of *CAL* and *EF-1α* were identified by aligning a representative sequence of each region by BLAST in the NCBI database (http://blast.ncbi.nlm.nih.gov/Blast.cgi). Searches were made using the blastn (for ITS) and blastx (for *CAL* and *EF-1α*) algorithms that search a nucleotide database and a protein database using a translated nucleotide query, respectively. The two best fungal species hits were aligned to each gene region and used to identify the conserved or coding regions for each locus. For *EF-1α* and *CAL*, translation of the coding regions was performed in Geneious v6. For the entire *E*. *maculosum* population, the number of segregating sites in each locus, the number of substitutions in the 5.8S rDNA, the number of synonymous substitutions and nonsynonymous substitutions in coding regions of *EF-1α* and *CAL*, and *d*_N_/*d*_S_ ratios for *EF-1α* and *CAL* were determined in DnaSP v5 [28].

### Phylogenetic analyses

Phylogenies were inferred for each locus using maximum likelihood (ML) in PAUP*4b v10 [29] and Bayesian inference (BI) in MrBayes v3.0 [30]. *E*. *rostrupii* was used as the outgroup. DT-ModSel [31] was used to estimate the best-fit nucleotide substitution models for each dataset. Robustness and support for clades for the ML phylogeny were assessed using 200 bootstraps. The Shimodaira-Hasegawa (SH) tests of topological congruence were conducted on the ML phylogenies as implemented in PAUP with RELL 1000 resampling replicates using modified datasets to include 76 isolates that contained data for all three gene regions that were included in the combined dataset and *E*. *rostrupii*. BI was performed with parameter settings suggested by the best-fit nucleotide substitution models. The Markov chain Monte Carlo (MCMC) search was run with four chains for 3,000,000 generations generating 30,001 trees; the first 6,000 trees were discarded as "burn-in" of the chains.

### Haplotype network, nucleotide diversity, and tests of neutrality and recombination

Haplotype networks were constructed for each locus using parsimony in the program TCS v1.21 with a 92% similarity cutoff [32]. Isolates were assigned to populations based on geographic origin including: *Exobasidium* from Maine and Nova Scotia (northeastern North America or NE), *E*. *maculosum* from Georgia and North Carolina (southeastern USA or SE), *E*. *maculosum* from Georgia (GA), *E*. *maculosum* from North Carolina (NC), and *E*. *maculosum* and *Exobasidium* from lowbush blueberry (NE+SE). The number of unique haplotypes, haplotype diversity (*h*_D_), pairwise nucleotide diversity (*π*) [33], Watterson’s Theta (*θ*_w_) [34], and the number of segregating sites (S) for each locus and the combined dataset in each population separately (NE, SE, GA, NC) and in the total population (Total) were estimated. Neutrality of each locus was estimated by Tajima’s *D* [35] and Fu and Li’s *F* [36] in DnaSP v5. Permutation tests, with 1000 randomizations, were used to test for significant departures from neutrality [37]. The minimum number of recombination events [38] that occurred for each locus and for the combined dataset within each geographically assigned population, were estimated using coalescent simulations within DnaSP v5.

### Divergence among populations

Two measures of genetic differentiation, *K*_ST_* [39] and *S*_nn_ [40] were used to determine if populations were structured by geographic origin (NE, *n* = 4; SE, *n* = 72; NC, *n* = 12; GA, *n* = 60), original host within the southeastern USA (rabbiteye, *n* = 57; southern highbush, *n* = 13; highbush, *n* = 2), or tissue type (fruit, *n* = 9 or leaf, *n* = 63, excluding NE isolates). The statistics were determined using the combined loci dataset. *K*_ST_* is estimated by determining the proportion of genetic diversity within populations compared to the diversity in the total population. Hudson’s nearest-neighbor statistic (*S*_nn_) is a measure of how frequently the most similar sequence or "nearest neighbor" is from the same assigned population. *S*_nn_ approaches one when populations are highly differentiated and near one-half when populations are panmictic [40]. Significance for both tests was estimated by 1000 permutations of the data as implemented in DnaSP v5.

## Results

### Genetic diversity

A total of 1775 nucleotides were sequenced for 82 isolates of *E*. *maculosum* from the southeastern USA, 6 isolates from *V*. *angustifolium* in northeastern North America, and an isolate of *E*. *rostrupii* from *V*. *macrocarpon* (cranberry) collected from New Jersey, USA that was used as an outgroup. Haplotype diversity (*h*_D_) was very high across all loci for all populations. In the combined dataset, each of the 76 isolates had unique multilocus haplotypes with a haplotype diversity (*h*_D_) of 1.00 (Table 2). The most diverse locus for the total population was *EF-1α* with nucleotide diversity (*π*) of 0.024 and 182 segregating sites among the 949 sequenced nucleotides (Table 2). However, *CAL* and ITS provided similar levels of diversity for the total population with *π* of 0.022 and 0.012, respectively. Significant levels of recombination were detected for *EF-1α*, with a minimum of 42 unique events (*R*_min_) occurring within the entire population. Lower levels of recombination were observed for ITS and *CAL* with 7 and 8 unique recombination events occurring within the combined population, respectively.

**Table 2:** Number of haplotypes, haplotype diversity, segregating sites, nucleotide diversity, population mutation rate, neutrality estimates, and minimum number of recombination events for each locus in the total population of *Exobasidium maculosum* collected from blueberry and from each geographic subpopulation.

Locus (nucleotides)	population^a^	haplotypes (*n*)^b^	*h*_D_ ^c^	*S* (*P*)^d^	*Θ*_w_ ^e^	π^f^	Tajima’s *D* ^g^	Fu and Li’s *F* ^g^	*R*min^h^
ITS (575)	Total	61 (85)	0.98	77 (68)	0.029	0.012	- 1.79*	- 4.45*	7
	NE	3 (4)	0.83	4 (4)	0.004	0.004	- 0.78	- 0.78	0
	SE	58 (81)	0.98	75 (67)	0.028	0.011	- 1.88*	- 4.09*	7
	GA	53 (69)	0.99	67 (59)	0.026	0.011	- 1.73	- 3.66*	6
	NC	11 (12)	0.99	25 (25)	0.015	0.011	- 1.32	- 1.57	1
*EF-1α* (949)	Total	70 (81)	0.99	182 (163)	0.038	0.024	- 1.02	- 2.19	42
	NE	5 (5)	1.00	41 (40)	0.020	0.021	- 0.18	- 0.15	2
	SE	65 (76)	0.99	160 (145)	0.034	0.023	- 0.87	- 2.29	38
	GA	51 (62)	0.99	138 (127)	0.031	0.023	- 0.73	- 2.33	35
	NC	14 (14)	1.00	91 (85)	0.030	0.030	- 0.56	- 0.83	21
*CAL* (251)	Total	52 (79)	0.98	40 (38)	0.033	0.022	- 0.93	- 1.22	8
	NE	6 (6)	1.00	13 (13)	0.023	0.025	0.68	0.44	3
	SE	46 (73)	0.97	33 (30)	0.027	0.021	- 0.63	- 1.32	5
	GA	37 (61)	0.96	31 (30)	0.027	0.021	- 0.67	- 1.28	5
	NC	12 (12)	1.00	14 (14)	0.020	0.02	0.11	0.55	5
Combined (1775)	Total	76 (76)	1.00	286 (259)	0.031	0.02	-	-	57
	NE	4 (4)	1.00	55 (54)	0.017	0.017	-	-	4
	SE	72 (72)	1.00	267 (244)	0.032	0.019	-	-	50
	GA	60 (60)	1.00	225 (207)	0.028	0.019	-	-	47
	NC	12 (12)	1.00	121 (116)	0.023	0.018	-	-	26

^a^ Populations consist of the total population of *E*. *maculosum* (*n* = 88), isolates from Nova Scotia, Canada and Maine, USA (NE, *n* = 6), isolates from the southeastern USA (SE, *n* = 82), and isolates from Georgia (GA, *n* = 68) and North Carolina (NC, *n* = 14).

^b^ Number of haplotypes and total number of individuals included in the analyses shown within parentheses

^c^ Haplotype diversity calculated in DnaSP (28)

^d^ Segregating sites and number of parsimonious sites shown within parentheses calculated in DnaSP

^e^ Watterson’s theta calculated in DnaSP

^f^ Nucleotide diversity calculated in DnaSP

^g^ Tajima’s *D* and Fu and Li’s *F* calculated in DnaSP. Values that significantly differ from neutrality (*P* ≤ 0.05) based on 1000 permutations of the data are indicated by an asterisk (*)

^h^ Minimum number of recombination events calculated in DnaSP

### Signatures of purifying selection

Tajima’s *D* and Fu and Li’s *F* were estimated for ITS, *EF-1α*, and *CAL* (Table 2) to determine if selection was acting on any of the loci or if demographic processes in the populations were affecting neutrality. Estimates for ITS were significantly negative (*p* ≤ 0.05), indicating positive selection at or near this locus. Most estimates for the other loci in each population were negative. The slightly negative, non-significant values observed at all other loci are indicative of purifying selection. Selection was further investigated by determining if substitutions within coding regions of *EF-1α* and *CAL* were synonymous or non-synonymous and if the 5.8S region between ITS1 and ITS2 was conserved. BLASTn analysis of the ITS nucleotide sequence from *E*. *maculosum* against the GenBank database resulted in two best hits of *E*. *inconspicuum* (e-value: 0.0, Genbank: ABG72749) and *E*. *arescens* (e-value: 0.0, Genbank: ABG53988). BLASTn analysis of *EF-1α* nucleotide sequence from *E*. *maculosum* against the GenBank database resulted in two best hits of *E*. *rhododendri* (e-value: 1 × 10^−171^, Genbank: ABG72749) and *E*. *gracile* (e value: 1 × 10^−170^, Genbank: ABG53988). Only two non-parsimonious segregating sites were observed within the 5.8S region among all isolates. The putative coding regions and translated amino acid sequences of *E*. *rhododendri* and *E*. *gracile* were used to determine the putative coding regions and amino acid sequence of *EF-1α* for *E*. *maculosum*. Out of the total 182 segregating sites within the coding region, 176 were synonymous, and only 6 resulted in an amino acid change (S1 Fig). Further, the putative coding regions and translated amino acid sequences of *CAL* from *Mortierella verticillata* (e-value: 7 × 10^−27^, Genbank: KFH70124) and *Rhizoctonia solani* AG-3 (e-value: 31 × 10^−27^, Genbank: EUC57218) were used to identify the putative coding regions and amino acid sequence for *CAL* in *E*. *maculosum*. Out of 40 segregating sites in the coding region, only 8 resulted in amino acid changes. The *d*_N_/*d*_S_ ratios for *EF-1α* and *CAL* were 0.03 and 0.20, respectively, which indicates that although nucleotide diversity is high, purifying selection is acting on these genes to conserve the amino acid sequence of the proteins.

### Phylogenetic analyses

**Fig 1:**
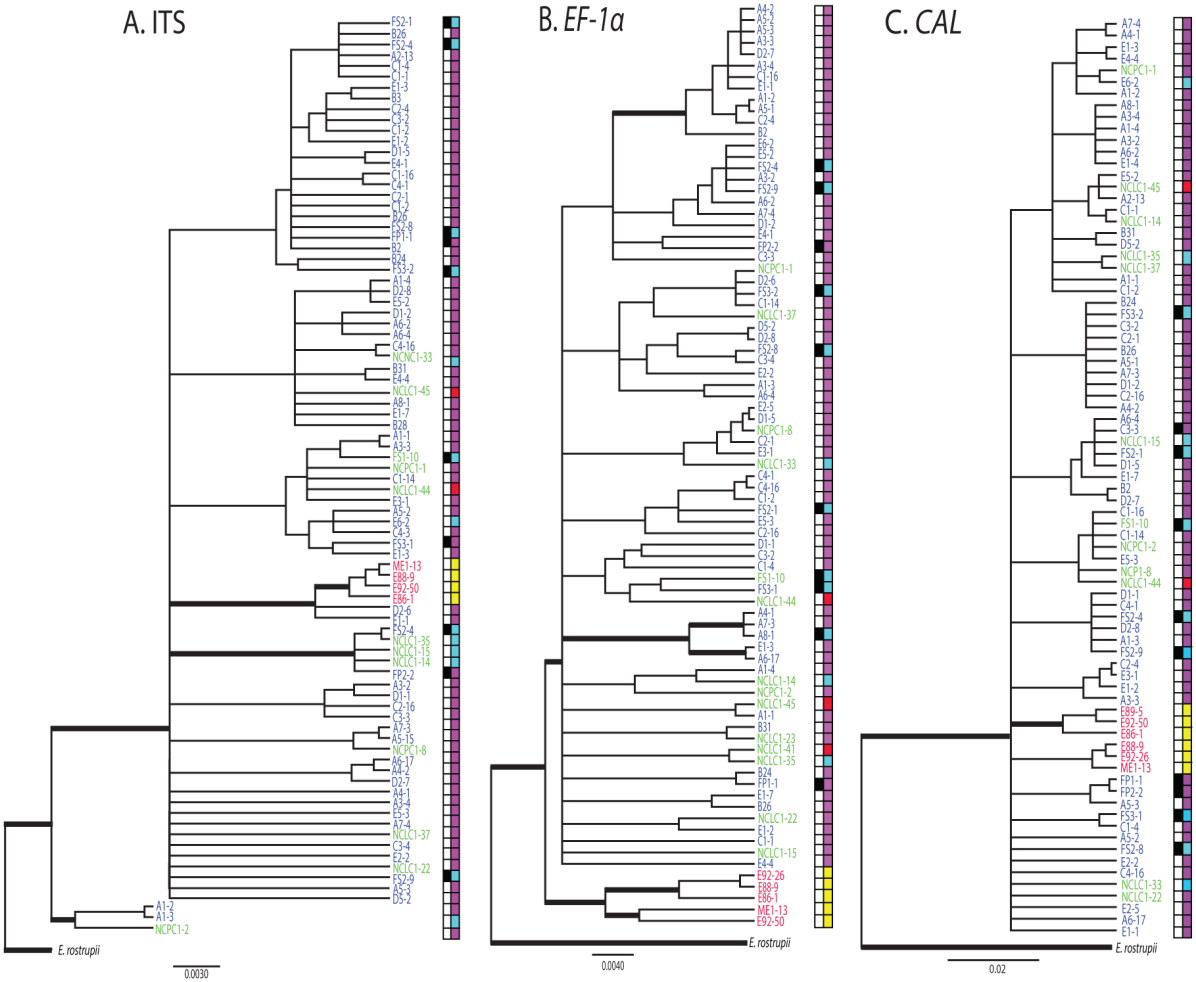
Bayesian inferred phylogenies for isolates of *Exobasidium maculosum* for the loci A. ITS, B. *EF-1α*, C. *CAL*. Phylogenies were rooted with *E*. *rostrupii*. Bold nodes indicate bootstrap support values obtained by maximum likelihood and Bayesian posterior probabilities greater than 70 and 0.90, respectively. Isolate names are colored by geographic location: blue = Georgia; green = North Carolina; red = northeastern North America. The first column of boxes to the right of each phylogeny indicates isolates that were collected from leaf (white) or fruit (black) plant tissues. The second column indicates the host from which isolates were collected: violet = rabbiteye blueberry (*Vaccinium virgatum*); blue = southern highbush blueberry (*Vaccinium corymbosum* hybrid); red = highbush blueberry (*V*. *corymbosum*); yellow = lowbush blueberry (*V*. *angustifolium*). Isolates of *E*. *rostrupii* were collected from leaf spots on cranberry (*V*. *macrocarpon*).

To determine if the *Exobasidium* population from lowbush blueberry in northeastern North America represents a distinct species, ML and BI phylogenies were generated for each of the three loci. The best-fit model of DNA evolution for ITS and *EF-1α* was equal-frequency Tamura-Nei with invariable sites and a gamma distribution (TrNef+I+G) [41], and for *CAL* was the generalized time reversible with a gamma distribution (GTR+G) [42]. Based on the results of the SH tests, the gene tree topologies were incongruent (*P* = 0.001), thus loci were not combined for phylogenetic analyses. For ITS, isolates from lowbush blueberry in northeastern North America (NE) clustered in a well-supported clade with 0.99 posterior probability (pp) and 100% bootstrap support (bs); however, two isolates from Georgia (GA) also clustered with this group (Fig 1A). Additionally, this clade was nested within a larger clade including most of the isolates from the southeastern USA (SE) making the SE population paraphyletic. For *EF-1α*, NE isolates formed a well-supported monophyletic clade (0.99 pp and 100% bs) (Fig 1B). For *CAL*, NE isolates were polyphyletic (Fig 1C). Only the *EF-1α* locus showed reciprocal monophyly for the NE and SE populations. Clade placement across loci was inconsistent among the SE isolates as well, indicating signatures of recombination or incomplete lineage sorting. For example, isolates FS2-4, NCLC1-35, NCLC1-14, NCLC1-15, and FP2-2 formed a well-supported clade for ITS, but these isolates were not clustered for *EF-1α* or *CAL* (Fig 1). Further, no consistent clustering occurred across all three loci for isolates collected from leaf or fruit spots, or from isolates collected from similar hosts (rabbiteye, southern highbush, and highbush). However, for ITS and *EF-1α* isolates from lowbush blueberry (NE) clustered into a single clade (Fig 1).

### Population structure and recombination

**Fig 2:**
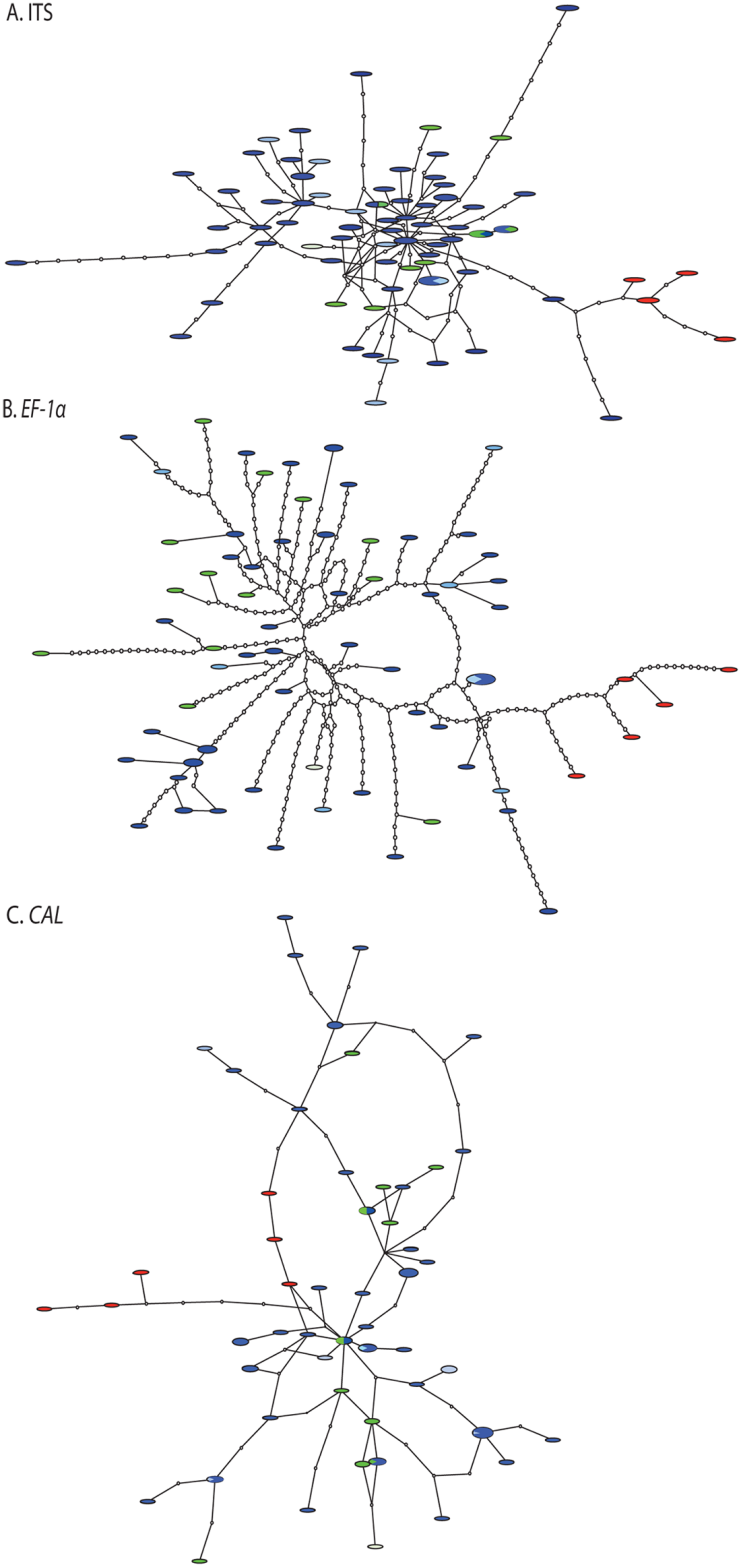
Multilocus haplotype networks (92% criterion) of *Exobasidium maculosum* isolates constructed in TCS v1.21 (32) for the loci A. ITS, B. *EF-1α*, C. *CAL*. Each haplotype is represented as a circle proportional in size to the number of isolates in each haplotype. Inferred intermediate haplotypes are represented by a small solid dot. Each line segment represents a single mutation. The number of isolates in each haplotype collected from Georgia (blue) and North Carolina (green) and leaf (dark color shades) and fruit (light color shades) plant tissue are proportionally represented in pie charts. Haplotypes in red represent isolates from northeastern North America.

Haplotype networks of the three loci highlighted the extreme genetic diversity in *E*. *maculosum*. Each network contained an extensive number of haplotypes with very few repeated haplotypes (Fig 2). The ITS and *EF-1α* networks (Fig 2A and 2B) revealed clustering of isolates from the NE, but a single cluster was not evident in the *CAL* network (Fig 2C). Significant genetic differentiation was detected (*P* < 0.001) between the NE and SE populations based on estimates of population structure *K*_ST_* and *S*_nn_ on the combined dataset (Table 3). However, genetic differentiation between GA and NC populations showed significant structure for only one of the estimates (*S*_nn_ = 0.810; *P* = 0.060, *K*_ST_* = 0.005; *P* < 0.006). Isolates collected from fruit and leaf spots were not genetically distinct based on gene trees, haplotype networks or estimates of population structure (Figs 1 and 2, Table 3).

**Table 3:** Measures of genetic differentiation for populations of *Exobasidium maculosum* collected from blueberry.

Populations compared^a^	*S*_nn_ ^b^	*K*_ST_*^b^
NE vs. SE	1.00 ^p < 0.001^	0.013 ^p < 0.001^
GA vs. NC	0.810	0.005 ^p < 0.006^
R vs. S vs. H	0.755	0.004
fruit vs. leaf	0.830	0.002

^a^ Geographic populations consist of isolates from Nova Scotia, Canada and Maine, USA (NE), isolates from the southeastern USA (SE), and isolates from Georgia (GA) and North Carolina (NC). Comparison of populations based on host species within SE include: *V*. *virgatum* or rabbiteye blueberry (R), *V*. *corymbosum* hybrid or southern highbush blueberry (S), *V*. *corymbosum* or highbush blueberry (H). Comparison of populations based on host tissue within SE include fruit spots and leaf spots

^**b**^ *S*_nn_ and *K*_ST_* calculated in DnaSP (28). Values that significantly differ from neutrality (*P* ≤ 0.05) based on 1000 permutations of the data are indicated

Abundant reticulation was present in the haplotype networks for all three loci, indicating recurrent recombination among the SE isolates. For *CAL*, reticulation was observed among isolates from the NE and SE indicating contemporary or historical recombination (Fig 2C). Estimates of minimal recombination events (*R*_min_) indicated recombination between the NE and SE populations within each locus (*R*_min_ = 42 and 8 for *EF-1α* and *CAL*, respectively) and within the combined dataset (*R*_min_ = 58). There was also extensive recombination within each population (NE, SE, GA, and NC) for each locus and the combined dataset (Table 2).

## Discussion

The extremely high genetic diversity observed for *E*. *maculosum* is uncommon for fungi and unexpected for an emerging pathogen. The two factors contributing to high genetic diversity include a high population mutation rate (*θ*_W_ = 0.031 for the combined dataset in the total population) and a high level of recombination within and among loci (*R*_min_ = 57 for the combined dataset in the total population). Within ITS, we identified 77 segregating sites for *E*. *maculosum*, whereas there are very few, if any, segregating sites for the ITS region for most fungal species [43,44]. In fact, ITS shows such little variation within most species of fungi that it was selected as the barcode sequence to identify individuals to the species level [45]. Though few examples exist, high diversity within ITS was observed for populations of the amphibian chytridiomycosis fungus, *Batrachochytrium dendrobatidis*, collected from South Korea and other parts of Asia where at least 50 ITS haplotypes have been identified [46]. Similar to ITS, we found uncommonly high genetic diversity for *E*. *maculosum* within the housekeeping genes *EF-1α* and *CAL*. Although the sequences were diverse, we found purifying selection was acting within the coding regions. Historically, synonymous substitutions that do not change the amino acid sequence of proteins were thought to have no functional consequences, and thus were neutral and of little biological significance [47]. Recent research has shown, however, that synonymous mutations can impact cellular processes including gene regulation, mRNA structure and processing, and protein synthesis, conformation and functionality [48]. Synonymous substitution rates can be locus specific, driven by changes in adaptive or relaxed selection affecting certain genes [49]. Selection, mutation, and drift can act on synonymous sites altering codon usage patterns of genes within an organism [50]. Mechanisms including faulty DNA repair machinery or repeat-induced point mutation (RIP) could be the source of the high diversity we observe in *E*. *maculosum* populations [51,52]. With the limited loci sequenced here it is not possible to tease apart the mechanism(s); however, genomic and population genomic tools will allow us to search the *E*. *maculosum* genome for the presence of DNA repair and RIP machinery and to identify if this high level of diversity occurs within segments of the genome or whether it is widespread. The extreme genetic diversity we observe in *E*. *maculosum* indicates that the species has a high evolutionary potential providing it with an adaptive advantage [53].

In addition to a high population mutation rate, recombination contributes to the high genetic diversity in *E*. *maculosum*. The number of recombination events that occurred both within and among loci for the entire population suggests that *E*. *maculosum* populations are outcrossing. Most *Exobasidium* species produce both sexually-derived basidiospores, through a thick-walled basidium that is formed on the tissues of their hosts, and asexually-derived, yeast-like conidia [54]. Sundström [55] showed that within the genus *Exobasidium* some species have a homothallic, or self-compatible, mating system, rather than a heterothallic, or self-incompatible, mating system. Single-conidial isolates of *E*. *vaccinii* and *E*. *japonicum* inoculated onto *V*. *vitis-idaea* and *Azalea indices*, respectively, formed the sexually derived basidia and basidiospores [55,56]. However, it is not currently known if all species within *Exobasidium* are homothallic. We know that *E*. *maculosum* undergoes sexual reproduction because it produces basidia and basidiospores [14,16], but we hypothesize that the high levels of recombination and genotypic diversity are the result of outcrossing within populations. However, successful inoculations of single-conidial isolates onto blueberry hosts and a better understanding of the lifecycle will help determine if *E*. *maculosum* is homothallic or heterothallic, as fungi with both homothallic and heterothallic mating systems have the ability to outcross [57,58].

*Exobasidium* species have been studied primarily at the genus level using morphological and molecular systematics [22,54,59,60]. To our knowledge this is the first study of an *Exobasidium* species at the population level, which has allowed for a better examination of the phylogeography and evolutionary history of *E*. *maculosum*. One aim of this research was to understand why Exobasidium leaf and fruit spot is emerging on commercial blueberry in the southeastern USA. Often, emerging diseases arise when pathogen populations become more aggressive, are found on novel hosts, or occur in new geographic locations [61]. When disease emergence results from the introduction of a pathogen into a new area or the development of novel genotypes, typically pathogen populations exhibit low genetic diversity or few genetically diverse non-recombining haplotypes [62]. In populations of *E*. *maculosum*, haplotype diversity reached the highest value possible (*h*_D_ = 1) with each isolate represented by a unique multilocus haplotype, demonstrating extremely high genetic diversity [63]. These results suggest that the emergence of Exobasidium leaf and fruit spot within the southeastern USA is not the result of a recently introduced population or the evolution of a more aggressive genotype. Rather, this high genetic diversity suggests that large populations of *E*. *maculosum* have been established in the southeastern USA for much longer than the recent emergence. Additionally, the geographic population structure detected within the southeastern USA suggests that *E*. *maculosum* has had a historical presence in this region. Causes for the recent emergence of this disease are not clear, but could be the result of increasing populations of blueberry hosts within North Carolina and Georgia. Acreage of commercial blueberry plantings within the southeastern USA has increased rapidly within the last 15 years. In 1999, the area of harvested blueberry in Georgia was 4,400 ha and increased to 5,463 ha in 2013, with a similar trend occurring in North Carolina (National Agricultural Statistics Service 1999, 2014). Another explanation for disease emergence is a change in the environment, such as increasing winter temperatures or greater precipitation. Since *E*. *maculosum* can exist in a yeast form, populations could have persisted for years on the leaf phylloplane or as endophytes of *Vaccinium* hosts without causing symptoms, but became pathogenic when environmental or host plant conditions became conducive. This switch from non-pathogen to pathogen has been hypothesized for fungi that are able to exist as both endophytes and pathogens, such as those collected from grasses [64,65].

To better understand the demographic history of this pathogen, we used the neutrality estimates Tajima’s *D* and Fu and Li’s *F* to determine if populations had undergone population bottlenecks or expansions. In populations where bottlenecks have occurred, there is a reduction in low-frequency polymorphisms that generates positive Tajima’s *D* values [66,67], whereas in populations that have recently expanded, values of Tajima’s *D* and Fu and Li’s *F* will be significantly negative across multiple loci, which is caused by an excess of recent mutations that produce a star phylogeny [68,69]. Tajima’s *D* and Fu and Li’s *F* estimates showed no evidence for population bottlenecks or expansions in *E*. *maculosum* as most estimates did not significantly deviate from neutrality. It has been shown, however, that inferring demographic history in the presence of mutation rate heterogeneity is difficult [70]. Overall, these results suggest that the population size of *E*. *maculosum* has remained constant further supporting our conclusion that disease emergence is the result of an environmental change or a change in the host population.

Exobasidium leaf and fruit spot disease is found in the eastern Canadian provinces of Nova Scotia, New Brunswick, Quebec, and Ontario [17] and Maine, USA on *V*. *angustifolium* and in the southeastern USA on commercially produced blueberry hosts including *V*. *virgatum*, *V*. *corymbosum*, and *V*. *corymbosum* hybrids [14]. *Exobasidium* collected from *V*. *angustifolium* in northeastern North America and *E*. *maculosum* from commercial species in the southeastern USA were genetically distinct [14], but it was not previously clear if these represented distinct species. The genealogical concordance phylogenetic species recognition (GCPSR) concept [19] is increasingly being employed to determine species groups among closely-related basidiomycete fungi [71–74]. Using GCPSR as our species concept, the lack of reciprocal monophyly for most gene trees for populations from northeastern North America (NE) and populations from the southeastern USA (SE) showed that these are genetically distinct populations of *E*. *maculosum*, but not distinct species. Well-supported reciprocal monophyly of the NE population was observed for *EF-1α* but not for *CAL* or ITS, possibly due to incomplete lineage sorting or hybridization. The haplotype network for *CAL* showed reticulation between the NE and SE population suggesting historic or contemporary recombination. Ongoing gene flow between populations may be preventing speciation between the populations, or the populations are undergoing speciation or have recently speciated, but not enough time has passed for reciprocal monophyly to occur for the majority of gene trees.

To better understand the emergence and spread of this disease we determined if populations of *E*. *maculosum* were genetically structured by original blueberry host species, host tissue (leaf or fruit), or geography. We found that the main driver of population structure in *E*. *maculosum* is geography. Within the southeastern USA, genetic differentiation was not detected based on original blueberry host species or host tissue type suggesting that these factors are not driving population structure. This is also supported by the gene trees and haplotype networks. However, significant differentiation was detected between *E*. *maculosum* populations from northeastern North America and the southeastern USA. The genetic structure between these populations was suggested by previous results [14]. Differences in blueberry host species in these regions could be contributing to population structure, but this is not clear because geography and host species are confounded when comparing northeastern North America and southeastern USA populations of *E*. *maculosum*. All isolates from northeastern North America were collected from lowbush blueberry (*V*. *angustifolium*), which is genetically distinct from the blueberry species sampled in the southeastern USA [75,76]. Interestingly, *K*_ST_* was significant between *E*. *maculosum* isolates from Georgia and North Carolina suggesting that geography is a main driver for population divergence within the southeastern USA and that there is limited dispersal of *E*. *maculosum* between these regions.

## Supporting Information

S1 Fig Amino acid alignment of a portion of the *EF-1α* of *Exobasidium maculosum*. Changes of amino acids at a single position are highlighted in red, and sites highlighted in shades of grey indicated amino acid variation.
(PDF)

## References

[1] Pimentel D, McNair S, Janecka J, Wightman J, Simmonds C, O’Connell C, et al Economic and environmental threats of alien plant, animal, and microbe invasions. Agric Ecosyst Environ. 2001;84: 1–20.

[2] Fry W, Birch P, Judelson H, Grünwald NJ, Danies G, Everts KL, et al Re-emerging *Phytophthora infestans* . Phytopathol.; 2015. 10.1094/PHYTO-01-15-0005-FI 25760519

[3] Smith K, Draper M, Simmons K, Bennett R, Hebbar P, Royer M, et al US preparations for potential introduction of Ug99 strains of wheat stem rust. Outlook Pest Management. 2009;20: 148–152.

[4] Mikaberidze A, McDonald BA, Bonhoeffer S. Can high-risk fungicides be used in mixtures without selecting for fungicide resistance? Phytopathology. 2014;104: 324–331. 10.1094/PHYTO-07-13-0204-R 24025048

[5] Lucas JA, Hawkins NJ, Fraaije BA. The evolution of fungicide resistance. Adv Appl Microbiol. 2015;90: 29–92. 10.1016/bs.aambs.2014.09.001 25596029

[6] Andersen PK, Cunningham AA, Patel NG, Morales FJ, Espstein PR, Daszak P. Emerging infectious diseases of plants: pathogen pollution, climate change and agrotechnology drivers. Trends Ecol Evol. 2004;19: 536–544. 10.1016/j.tree.2004.07.021 16701319

[7] Fontaine MC, Austerlitz F, Giraud T, Labbe F, Papura D, Richard-Cervera S, et al Genetic signature of a range expansion and leap-frog event after the recent invasive of Europe by the grapevine downy mildew pathogen *Plasmopara viticola* . Mol Ecol. 2013;22: 2771–2786. 10.1111/mec.12293 23506060

[8] Gladieux P, Feurtey A, Hood ME, Snirc A, Clavel J, Dutech C, et al The population biology of fungal invasions. Mol Ecol. 2015;24: 1969–1986. 10.1111/mec.13028 25469955

[9] Brewer MT, Milgroom MG. Phylogeography and population structure of the grape powdery mildew fungus, *Erysiphe necator*, from diverse *Vitis* species. BMC Evol Biol. 2010;25: 268. 10.1186/1471-2148-10-268 PMC2941690 20809968

[10] Genton BJ, Shykoff JA, Giraud T. High genetic diversity in French invasive populations of common ragweed, *Ambrosia artemisiifolia*, as a result of multiple sources of introduction. Mol Ecol. 2005;14: 4275–4285. 16313592 10.1111/j.1365-294X.2005.02750.x

[11] Stukenbrock EH, Croll D. The evolving fungal genome. Fungal Biol Rev. 2014;28: 1–12.

[12] Chakraborty S. Migrate or evolve: options for plant pathogens under climate change. Glob Chang Biol. 2013;19: 1985–2000. 10.1111/gcb.12205 23554235

[13] Grünwald NJ, Goss EM. Evolution and population genetics of exotic and re-emerging pathogens: novel tools and approaches. Annu Rev Phytopathol. 2011;49: 249–267. 10.1146/annurev-phyto-072910-095246 21370974

[14] Brewer MT, Turner AN, Brannen PM, Cline WO, Richardson EA. *Exobasidium maculosum*, a new species causing leaf and fruit spots on blueberry in the southeastern USA and its relationship with other *Exobasidium* spp. parasitic to blueberry and cranberry. Mycologia. 2014;106: 415–423. 10.3852/13-202 24871592

[15] Smith B. Exobasidium leaf and fruit spot. Mississippi *Vaccinium* J. 2012;1: 10–11.

[16] Cline W. An Exobasidium disease of fruit and leaves of highbush blueberry. Plant Dis. 1998;82: 1064. 10.1094/PDIS.1998.82.9.1064B 30856844

[17] Nickerson NL, Vander Kloet SP. Exobasidium leaf spot of lowbush blueberry. Can J Plant Pathol. 1997;19: 66–68.

[18] Burt EA. The Thelophoraceae of North America IV. *Exobasidium* . Ann Mo Bot Gard. 1915;2: 627–658.

[19] Taylor JW, Jacobson DJ, Kroken S, Kasuga T, Geiser DM, Hibbett DS, et al Phylogenetic species recognition and species concepts in fungi. Fungal Genet Biol. 2000;31: 21–32. 11118132 10.1006/fgbi.2000.1228

[20] Chandramouli B. Blister blight of tea: biology, epidemiology and management. Annu Rev Plant Pathol. 2003;2: 145–162.

[21] Nagao H, Exuka A, Harada Y, Sato T. Two new species of *Exobasidium* causing Exobasidium disease on *Vaccinium* spp. in Japan. Mycoscience. 2006;47: 277–283.

[22] Nannfeldt JA. *Exobasidium*, a taxonomic reassessment applied of the European species. Symb Bot Uppsala. 1981;23: 1–72.

[23] Mims CW, Richardson EA. An ultrastructural study of the asexual spores of the plant pathogenic fungus *Exobasidium vaccinii* . Bot Gaz. 1987;148: 228–234.

[24] Pelczar MJ Jr, Reid RD. Laboratory exercises in microbiology. New York: McGraw-Hill Book Company; 1958 pp. 45–47.

[25] Mahuka GS. A simple extraction method suitable for PCR-based analysis of plant, fungal, and bacterial DNA. Plant Mol Biol Reporter. 2004;22: 71–81.

[26] White TJ, Bruns T, Lee S, Taylor JW. Amplification and direct sequencing of fungal ribosomal RNA genes for phylogenetics In: Innis MA, Gelfand DH, Sninsky JJ, White TJ, editors. PCR Protocols: A Guide to Methods and Applications. New York: Academic Press, Inc.; 1990 pp. 315–322.

[27] Carbone I, Kohn LM. A method for designing primer sets for speciation studies in filamentous ascomycetes. Mycologia. 1999;1: 553–556.

[28] Rozas JJ, Sanchez-DelBarrio JC, Messeguer X, Rozas R. DnaSP, DNA polymorphism analyses by the coalescent and other methods. Bioinformatics. 2003;19: 2496–2497. 14668244 10.1093/bioinformatics/btg359

[29] Swofford DL. PAUP*. Phylogenetic Analysis Using Parsimony (*and Other Methods). Version 4. Sunderland, MA: Sinauer Associates; 2003.

[30] Huelsenbeck JP, Ronquist F. MrBayes: Bayesian inference of phylogenetic trees. Bioinformatics. 2001;17: 754–755. 11524383 10.1093/bioinformatics/17.8.754

[31] Minin V, Abdo Z, Joyce P, Sullivan J. Performance-based selection of likelihood models for phylogeny estimation. Syst Biol. 2003;52: 674–683. 14530134 10.1080/10635150390235494

[32] Clement M, Posada D, Crandall KA. TCS: a computer program to estimate gene genealogies. Mol Ecol. 2000;9: 1657–1660. 11050560 10.1046/j.1365-294x.2000.01020.x

[33] Nei M. Molecular Evolutionary Genetics. New York: Columbia Univ. Press: 1987.

[34] Watterson G. On the number of segregating sites in the genetic model without recombination. Theor Pop. Biol. 1975;7: 256–276. 1145509 10.1016/0040-5809(75)90020-9

[35] Tajima F. The effect of change in population size on DNA polymorphism. Genetics. 1989;123: 597–601. 2599369 10.1093/genetics/123.3.597 PMC1203832

[36] Fu YX, Li WH. Statistical tests of neutrality of mutations. Genetics. 1993;133: 693–709. 8454210 10.1093/genetics/133.3.693 PMC1205353

[37] Hudson RR. Gene genealogies and the coalescent process. Oxf. Surv. Evol. Biol. 1990;7:1–44.

[38] Hudson RR, Kaplan NL. Statistical properties of the number of recombination events in the history of a sample of DNA sequences. Genetics. 1985;111: 147–164. 4029609 10.1093/genetics/111.1.147 PMC1202594

[39] Hudson RR, Boos DD, Kaplan NL. A statistical test for detecting population subdivision. Mol. Biol. Evol. 1992; 138–151. 1552836 10.1093/oxfordjournals.molbev.a040703

[40] Hudson RR. A new statistic for detecting genetic differentiation. Genetics. 2000;155: 2011–2014. 10924493 10.1093/genetics/155.4.2011 PMC1461195

[41] Tamura K, Nei M. Estimation of the number of nucleotide substitutions in the control region of mitochondrial DNA in humans and chimpanzees. Mol Biol Evol. 1993;10: 512–526. 8336541 10.1093/oxfordjournals.molbev.a040023

[42] Tavaré S. Some Probabilistic and Statistical Problems in the Analysis of DNA Sequences. Lectures on Mathematics in the Life Sciences (American Mathematical Society) 1986;17: 57–86.

[43] Anderson IC, Campbell CD, Prosser JI. Potential bias of fungal 18S rDNA and internal transcribed spacer polymerase chain reaction for estimating fungal biodiversity in soil. Environ Microbiol. 2003;5: 36–47. 12542711 10.1046/j.1462-2920.2003.00383.x

[44] Toju H, Tanabe AS, Yamamoto S, Sato H. High-coverage ITS primers for the DNA-based identification of ascomycetes and basidiomycetes in environmental samples. PLOS ONE. 2012;7: e40863 10.1371/journal.pone.0040863 22808280 PMC3395698

[45] Schoch CL, Seifert KA, Huhndorf S, Robert V, Spouge L, Levesque CA, et al Nuclear ribosomal internal transcribed spacer (ITS) region as a universal DNA barcode marker for Fungi. Proc Natl Acad Sci USA. 2012;16: 6241–6246. 10.1073/pnas.1117018109 PMC3341068 22454494

[46] Bataille A, Fong JJ, Cha M, Wogan GO, Baek HJ, Lee H, et al Genetic evidence for a high diversity and wide distribution of endemic strains of the pathogenic chytrid fungus *Batrachochytrium dendrobatidis* in wild Asian amphibians. Mol Ecol. 2013;22: 4196–4209. 10.1111/mec.12385 23802586

[47] Chamary JV, Hurst LD. The price of silent mutations. Sci Am. 2009;300: 46–53. 10.1038/scientificamerican0609-46 19485088

[48] Hunt RC, Simhadri VL, Iandoli M, Sauna ZE, Kimchi-Sarfaty C. Exposing synonymous mutations. Trends Genet. 2014;30: 308–321. 10.1016/j.tig.2014.04.006 24954581

[49] Chamary JV. Hearing silence: Non-neutral evolution at synonymous sites in mammals. Nature Rev Gen. 2006;7: 98–108. 10.1038/nrg1770 16418745

[50] Hershberg R, Petrov DA. Selection on codon bias. Rev Genet. 2008;42: 287–299. 10.1146/annurev.genet.42.110807.091442 18983258

[51] Galagan JE, Selker EU. RIP: the evolutionary cost of genome defense. Trends Genet. 2004;20: 417–423. 15313550 10.1016/j.tig.2004.07.007

[52] Symington LS. DNA repair: making the cut. Nature. 2014;514: 39–40. 10.1038/nature13751 25231858

[53] McDonald BA, Linde C. Pathogen population genetics, evolutionary potential, and durable resistance. Annu Rev Phytopathol. 2002;40: 349–379. 12147764 10.1146/annurev.phyto.40.120501.101443

[54] Begerow D, Schäfer AM, Kellner R, Yurkov A, Kemler M, Oberwinkler F, et al Ustilaginomycotina In: McLaughlin DJ, Spatafora JW, editors. The Mycota. vol. 7. Part A. Systematics and Evolution, 2nd ed Berlin: Springer; 2014.

[55] Sundström KR. Studies of the physiology, morphology and serology of *Exobasidium* . Symb Bot Ups. 1964;18: 3, 89.

[56] Graafland W. The parasitism of *Exobasidium japonicum* Shir. on Azalea. Acia Bontanica Neerlandica. 1960;1: 347–379.

[57] Turgeon BG. Application of mating type gene technology to problems in fungal biology. Annu Rev Phytopathol. 1998;36: 115–137. 15012495 10.1146/annurev.phyto.36.1.115

[58] Billiard S, López-Villavicencio M, Hood ME, Giraud T. Sex, outcrossing and mating types: unsolved questions in fungi and beyond. J Evol Biol. 2012;25: 1020–1038. 10.1111/j.1420-9101.2012.02495.x 22515640

[59] Begerow D, Bauer R, Oberwinkler F. The Exobasidiales: an evolutionary hypothesis. Mycol Prog. 2002;1: 187–199.

[60] Piątek M, Lutz M, Welton P. *Exobasidium darwinii*, a new Hawaiian species infecting endemic *Vaccinium reticulatum* in Haleakala National Park. Mycol Prog. 2012;11: 361–371.

[61] Giraud T, Gladieux P, Gavrilets S. Linking the emergence of fungal plant diseases with ecological speciation. Trends Ecol Evol. 2010;25: 387–395. 10.1016/j.tree.2010.03.006 20434790 PMC2885483

[62] Milgroom MG. Recombination and the multilocus structure of fungal populations. Annu Rev Phytopathol. 1996;34: 457–478. 15012552 10.1146/annurev.phyto.34.1.457

[63] Nei M, Tajima F. DNA polymorphism detectable by restriction endonucleases. Genetics. 1981;97: 145–163. 6266912 10.1093/genetics/97.1.145 PMC1214380

[64] Eaton CJ, Cox MP, Scott B. What triggers grass endophytes to switch from mutualism to pathogenism? Plant Sci. 2001;180: 190–195. 10.1016/j.plantsci.2010.10.002 21421360

[65] Van Kan JAL, Shaw MW, Grant-Downton RT. *Botrytis* species: relentless necrotrophic thugs or endophytes gone rogue. Mol Plant Pathol. 2014;15: 957–961. 10.1111/mpp.12148 24754470 PMC6638755

[66] Nei M, Maruyama T, Chakraborty R. The bottleneck effect and genetic variability in populations. Evolution 1975;29: 1–10. 28563291 10.1111/j.1558-5646.1975.tb00807.x

[67] Gattepaille LM, Jakobsson M, Blum MGB. Inferring population size changes with sequence and SNP data: lessons from human bottlenecks. Heredity. 2013;110: 409–419. 10.1038/hdy.2012.120 23423148 PMC3630807

[68] Biswas S, Akey JM. Genomic insights into positive selection. Trends Genet. 2006;22: 437–446. 16808986 10.1016/j.tig.2006.06.005

[69] Alexandrino J, Arntzen JW, Ferrand N. Nested clade analysis and the genetic evidence for population expansion in the phylogeography of the golden-striped salamander, *Chioglossa lusitanica* (Amphibia: Urodela). Heredity. 2002;88: 66–74. 11813109 10.1038/sj.hdy.6800010

[70] Aris-Brosou S, Excoffier L. The impact of population expansion and mutation rate heterogeneity on DNA sequence polymorphism. Mol Biol Evol. 1996;13: 494–504. 8742638 10.1093/oxfordjournals.molbev.a025610

[71] Shen Q, Geiser DM, Royse DJ. Molecular phylogenetic analysis of *Grifola frondosa* (maitake) reveals a species partition separating eastern North American and Asian isolates. Mycologia. 2002;94: 472–482. 21156518

[72] Kauserud H, Stensrud Ø, Decock C, Shalchian-Tabrizi K, Schumacher T. Multiple gene genealogies and AFLPs suggest cryptic speciation and long-distance dispersal in the basidiomycete *Serpula himantioides* (Boletales). Mol Ecol. 2006;15: 421–431. 16448410 10.1111/j.1365-294X.2005.02768.x

[73] Harder CB, Læssøe T, Frølev TG, Ekelund F, Rosendahl S, Kjøller R. A three-gene phylogeny of the *Mycena pura* complex reveals 11 phylogenetic species and shows ITS to be unreliable for species identification. Fungal Biol. 2013;117: 764–775. 10.1016/j.funbio.2013.09.004 24295915

[74] Vialle A, Feau N, Frey P, Bernier L, Hamelin RC. Phylogenetic species recognition reveals host-specific lineages among poplar rust fungi. Mol Phylogenet Evol. 2013;66: 628–644. 10.1016/j.ympev.2012.10.021 23147268

[75] Levi A, Rowland LJ. Identifying blueberry cultivars and evaluating their genetic relationships using randomly amplified polymorphic DNA (RADP) and simple sequence repeat- (SSR-) anchored primers. J Am Soc Hort Sci. 1997;122: 74–78.

[76] Bell DJ, Rowland LJ, Polashock JJ, Drummond FA. Suitability of EST-PCR markers developed in highbush blueberry for genetic fingerprinting and relationship studies in lowbush blueberry and related species. J Am Soc Hort Sci. 2008;133: 701–707.

