# Supplementary material for: Elevated Genetic Diversity in the Emerging Blueberry Pathogen Exobasidium maculosum
Source: PLoS One. 2015 Jul 24;10(7):e0132545. doi: 10.1371/journal.pone.0132545 (PMC4514876; doi:10.1371/journal.pone.0132545)
Supplement: S1 Fig — Changes of amino acids at a single position are highlighted in red, and sites highlighted in shades of grey indicated amino acid variation. (PDF) [file pone.0132545.s001.pdf]

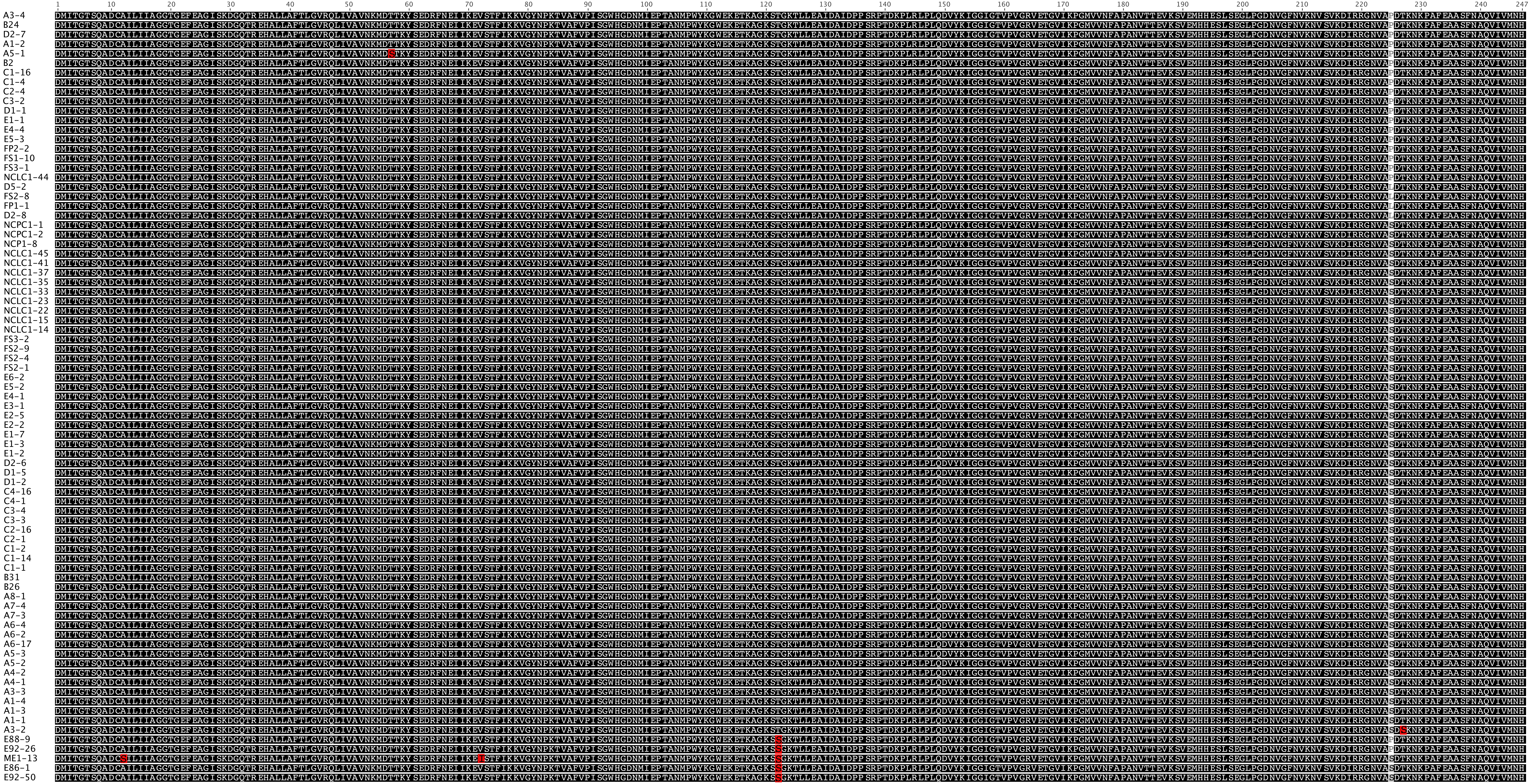

1
10
20
30
40
50
60
70
80
90
100
110
120
130
140
150
160
170
180
190
200
210
220
230
240
247
A3-4
B24
D2-7
A1-2
A5-1
B2
C1-16
C1-4
C2-4
C3-2
D1-1
E1-1
E4-4
E5-3
FP2-2
FS1-10
FS3-1
NCLC1-44
D5-2
FS2-8
FP1-1
D2-8
NCPC1-1
NCPC1-2
NCP1-8
NCLC1-45
NCLC1-41
NCLC1-37
NCLC1-35
NCLC1-33
NCLC1-23
NCLC1-22
NCLC1-15
NCLC1-14
FS3-2
FS2-9
FS2-4
FS2-1
E6-2
E5-2
E4-1
E3-1
E2-5
E2-2
E1-7
E1-3
E1-2
D2-6
D1-5
D1-2
C4-16
C4-1
C3-4
C3-3
C2-16
C2-1
C1-2
C1-14
C1-1
B31
B26
A8-1
A7-4
A7-3
A6-4
A6-2
A6-17
A5-3
A5-2
A4-2
A4-1
A3-3
A1-4
A1-3
A1-1
A3-2
E88-9
E92-26
ME1-13
E86-1
E92-50
